# EEG alpha activity increased in response to transcutaneous electrical nervous stimulation in young healthy subjects but not in the healthy elderly

**DOI:** 10.7717/peerj.8330

**Published:** 2020-01-07

**Authors:** Ebru Yıldırım, Bahar Güntekin, Lütfü Hanoğlu, Candan Algun

**Affiliations:** 1 Department of Physical Therapy and Rehabilitation/Graduate School of Health Sciences, Istanbul Medipol University, Istanbul, Turkey; 2 Department of Biophysics/School of Medicine, Istanbul Medipol University, Istanbul, Turkey; 3 REMER, Clinical Electrophysiology, Neuroimaging, and Neuromodulation Lab., Istanbul Medipol University, Istanbul, Turkey; 4 Department of Neurology/School of Medicine, Istanbul Medipol University, Istanbul, Turkey; 5 Department of Physical Therapy and Rehabilitation/School of Health Sciences, Istanbul Medipol University, Istanbul, Turkey; 6 Department of Orthesis-Prosthesis/School of Health Sciences, Istanbul Medipol University, Istanbul, Turkey

**Keywords:** Pain, TENS, EEG, FFT, Alpha

## Abstract

Transcutaneous Electrical Nerve Stimulation (TENS) is used not only in the treatment of pain but also in the examination of sensory functions. With aging, there is decreased sensitivity to somatosensory stimuli. It is essential to examine the effect of TENS application on the sensory functions in the brain by recording the spontaneous electroencephalogram (EEG) activity and the effect of aging on the sensory functions of the brain during the application. The present study aimed to investigate the effect of the application of TENS on the brain’s electrical activity and the effect of aging on the sensory functions of the brain during application of TENS. A total of 15 young (24.2 ± 3.59) and 14 elderly (65.64 ± 4.92) subjects were included in the study. Spontaneous EEG was recorded from 32 channels during TENS application. Power spectrum analysis was performed by Fast Fourier Transform in the alpha frequency band (8–13 Hz) for all subjects. Repeated measures of analysis of variance was used for statistical analysis (*p* < 0.05). Young subjects had increased alpha power during the TENS application and had gradually increased alpha power by increasing the current intensity of TENS (*p* = 0.035). Young subjects had higher alpha power than elderly subjects in the occipital and parietal locations (*p* = 0.073). We can, therefore, conclude that TENS indicated increased alpha activity in young subjects. Young subjects had higher alpha activity than elderly subjects in the occipital and somatosensory areas. To our knowledge, the present study is one of the first studies examining the effect of TENS on spontaneous EEG in healthy subjects. Based on the results of the present study, TENS may be used as an objective method for the examination of sensory impairments, and in the evaluative efficiency of the treatment of pain conditions.

## Introduction

Transcutaneous Electrical Nerve Stimulation (TENS) is a non-pharmacological intervention, commonly used in the treatment of chronic and acute pain conditions in physical therapy and rehabilitation. It is based on the principle of applying an alternating current to the skin from a battery-powered device through the electrodes placed on the skin (Vance et al., 2014). The mechanism of TENS in pain relief is not known, but several theories have been proposed. The first theory is the gate control theory of pain proposed by Melzack & Wall (1965). According to gate control theory, when afferent nerve fibers are stimulated, the pathway for painful stimuli is blocked by the operation of the gate at the level of the medulla spinalis resulting in the inhibition of pain conduction. The second theory is the endogenous opioid theory. According to the second theory, painful stimuli cause chemical changes in the brain, especially the release of pain-related opioids. These opioids (β-endorphin, met-enkephalins, leu-enkephalins, and the dynorphins) act as neuromodulators and neurotransmitters on three main classes of receptors and have an analgesic effect (Holden, Jeong & Forrest, 2005; Dowswell et al., 2009).

Pain sense starts from nociceptive receptors and travels from the medulla spinalis to the thalamus via spinothalamic tracts. Finally, from the thalamus, it travels to the somatosensory cortical area via the thalamocortical fibers. Therefore, the thalamus, thalamocortical fibers, spino-thalamic tracts, and nociceptors are essential anatomical structures in the transmission of pain (Wickremaratchi & Llewelyn, 2006; Price, 2002). Positron emission tomography (PET) scan studies showed that there was activation in the sensorial cortex, other parts of parietal cortex and occipital cortex during painful stimuli (Aydın, 2002). Furthermore, lesion studies and functional magnetic resonance imaging (fMRI) studies demonstrated that the application of painful stimuli caused activation in the thalamus, primary somatosensory cortex, secondary somatosensory cortex, and posterior parietal cortex (Price, 2002; Porro et al., 2002).

The central nervous system is important in the perception of pain. Central nervous system activation is associated with the processing of painful stimuli and is decreased in elderly subjects (Wickremaratchi & Llewelyn, 2006). The structure of the central nervous system and peripheral nervous system changes with aging. These changes in the central nervous system may be the result of a slowing in the processing of sensory stimuli, most likely due to the reduction in the speed of sensory nerve conduction (Victor & Ropper, 2001; Katzman & Terry, 1983). With age, there is a decrease in the speed of sensory conduction and the amplitude of the action potential resulting from sensory stimulation (Verdú et al., 2000). Most studies have investigated the sensory threshold of young and elderly subjects. Kenshalo (1986), compared sensory thresholds by using cutaneous stimulation in young and elderly subjects and observed that elderly subjects were less sensitive to sensory stimuli. In the literature, a loss of sensory acuity of the elderly is reported (Wickremaratchi & Llewelyn, 2006).

Electroencephalogram (EEG) is an electrophysiological technique used for the recording of brain electrical activity (Britton et al., 2016). In the literature, there are many studies which investigated the pain-related EEG responses and brains’ electrical activity during the modulation of pain by changes in stimulus intensity (e.g., low intensity and/or high-intensity stimuli) (Chen & Rappelsberger, 1994; Chang et al., 2003; Babiloni et al., 2006; Dowman, Rissacher & Schuckers, 2008; Egsgaard, Wang & Arendt-Nielsen, 2009; Nir et al., 2012; Tiemann et al., 2015; Li et al., 2016; Misra et al., 2017; Wang et al., 2019; Vuckovic et al., 2019). Most of these studies demonstrated that mainly alpha frequency was (alpha power) related to the intensity of pain perception (Le Pera et al., 2000; Chang et al., 2001, 2003; Babiloni et al., 2006; Egsgaard, Wang & Arendt-Nielsen, 2009; Tiemann et al., 2015; Li et al., 2016; Tu et al., 2016; Wang et al., 2019; Vuckovic et al., 2019). Tiemann et al. (2015) applied painful stimulation to the dorsal face of the hand. These authors demonstrated that alpha responses were significantly higher in the high-intensity condition than in the low-intensity condition. In the studies analyzing perception of pain “thermal stimulation (hot and cold),” “pressure,” “intramuscular injection,” and “laser” were used. The applications listed above are all somatosensory stimulations causing pain. However, the preliminary aim of the present study is not to produce pain but find out the effects of TENS application, which is a device mostly used in the treatment of pain. There are few studies which have analyzed the effect of TENS with EEG (Olson, 1993; Urasaki et al., 1998; Hoshiyama & Kakigi, 2000; Silva et al., 2014; Pierleoni, Gizdulich & Paoletti, 2011; Ebrahimian et al., 2018). In these studies EEG was recorded to find out the pre and post-treatment effects of TENS application in different groups of subjects. None of the studies has analyzed the direct effects of TENS on central nervous system by application of TENS and EEG recordings simultaneously in young and elderly subjects. Since TENS device is commonly used to treat the pain in elderly patients in different clinics, it is essential to know if the effect of TENS in the central nervous system will be the same in healthy young group (HY) and healthy elderly group (HE).

The investigation of the alpha frequency is important for understanding their role in sensory functions in humans. In the literature, there are several EEG indices (e.g., alpha peak frequency, alpha bandwidth, and amplitude) used for evaluation of the spontaneous EEG alpha activity (Bazanova & Vernon, 2014). We have taken into account the alpha frequency peak and alpha bandwidth which are alpha indices in our study because previous studies reported that this indices changes based on age or stimuli (Stroganova, Orekhova & Posikera, 1999; Bazanova, 2008; Samuel et al., 2018). There are also many EEG and magnetoencephalography (MEG) studies which were evaluated evoked responses (Golding et al., 1986; Olson, 1993; Tinazzi et al., 1997, 2000; Urasaki et al., 1998; Rossi et al., 1998, 2003; Hoshiyama & Kakigi, 2000; Johnson, Oxberry & Simpson, 2008; Passmore, Murphy & Lee, 2014; Peng et al., 2019). Researchers analyzed evoked responses components (somatosensory evoked potentials (SEPs) and somatosensory evoked fields (SEFs)) during somatosensory stimulations (e.g., TENS). In these studies the authors reported that N20, P27 (P22 or P25), N30 components of SEP were significantly increased with the presence of pain. When TENS was applicated these pain-related responses were decreased (Urasaki et al., 1998; Hoshiyama & Kakigi, 2000; Peng et al., 2019). Hoshiyama & Kakigi (2000) investigated the effect of TENS application by using EEG and MEG. SEP and SEF were recorded before and after TENS. The authors reported that N150 and P220 responses, which are SEP components were decreased post-TENS sessions compared to pre-TENS sessions. However, they reported that there was no considerable change in SEF components (1 M (90 ms) and 2 M (160 ms)) in post-TENS sessions compared to pre-TENS sessions.

There are some difficulties due to multiple comorbidities and polypharmacy in the treatment of pain in elderly individuals. Previous studies have reported that TENS application, which is given in different current intensities (low-intensity and high-intensity) decreased pain (Bjordal, Johnson & Ljunggreen, 2003; Johnson & Martinson, 2007; Claydon et al., 2011; Bergeron-Vézina et al., 2015). This beneficial effect of TENS was important in the nonpharmacological treatment of the pain in the elderly subjects who prone to pharmacological side-effects (Chabal et al., 1998; Elvir-Lazo & White, 2010). Most of the studies which evaluated the clinical efficacy of TENS were on young subjects. There was a limited number of studies, which have investigated the effect of aging on TENS application (Grant et al., 1999; Cheing, Hui-Chan & Chan, 2002; Ng, Leung & Poon, 2003; Bergeron-Vézina et al., 2015; Daguet et al., 2018; Bretherton et al., 2019). Bergeron-Vézina et al. (2015) reported TENS as an effective nonpharmacological treatment option in young, but not in elderly subjects. None of the studies has analyzed the direct effects of TENS on central nervous system by application of TENS and EEG recordings simultaneously in elderly subjects. Since TENS device is commonly used to treat the pain in elderly patients in different clinics, it is essential to know if the effect of TENS in the central nervous system will be the same in HY and HE.

The research questions addressed in this study are:

(1) Is the spontaneous EEG activity affected by the different current intensities of (minimum, medium, and maximum) TENS?
(2) What is the influence of aging on spontaneous EEG activity recorded in the different current intensities of TENS?

Since alpha frequency is associated with sensory functions, we have hypothesized that the alpha activity would increase in response to TENS used as somatosensory stimuli. The spontaneous EEG alpha activity would be differently sensitive to different intensities of the current. Furthermore, we hypothesized that as sensory functions are influenced by aging, the alpha activity induced by TENS application in the elderly subjects would be lower in comparison with young healthy subjects. To our knowledge, there is no previous study that has analyzed the effect of the different current intensities of TENS on the brains’ electrical activity by recording spontaneous EEG. In addition, the present study is one of the first to examine the effect of TENS on spontaneous EEG in healthy young and elderly subjects.

## Materials and Methods

### Subjects

A total of 29 healthy subjects were included in the study. Subjects were divided into two groups-healthy young and healthy elderly. The HY consisted of 15 participants between the ages of 18 and 30 who had no sensory loss, neurological or psychiatric disease. The HE consisted of 14 participants between the ages of 60 and 80 who had no sensory loss, neurological or psychiatric disease and who are. All subjects had normal sensory functions during a sharp/blunt test. Participants who used medications or substances that could affect the cognitive functions were not included; subjects for which TENS application is contraindicative were also excluded from the study.

The mean age was 24.2 years (SD 3.59) for the HY, and 65.64 years (SD 4.92) for the HE. There were eight women and seven men in the HY, and six women and eight men in the HE. There were 13 right-handed and two left-handed participants in the HY, and all participants in the HE were right-handed. The general demographic characteristics of the groups are shown in Table 1. The study was approved by The Non-Interventional Clinical Research Ethics Committee of Istanbul Medipol University (Ethical Report No: 10840098-604.01.01−E.6805). All participants signed an informed consent form.

**Table 1:** Demographic information of the groups.

	HY (*N* = 15) *M* ± SD	HE (*N* = 14) *M* ± SD
Age (year)	24.2 ± 3.59	65.64 ± 4.92
Gender (F/M)	8/7	6/8
Dominant hand (Right/Left)	13/2	14/0

**Note:**

F, Female; M, Male; *M*, Mean; SD, Standard Deviation; HY, Healthy young group; HE, Healthy elderly group.

### Stimuli and experimental procedure

Transcutaneous Electrical Nerve Stimulation was used as a somatosensory stimulus in the present study. First, the participants’ threshold of the perception of the electrical stimuli was evaluated. Electrical currents were applied to subjects without causing any muscle contractions or sensation of tingling. The subjects were asked to indicate their first sensation of electrical current. This value of the electrical current was identified as the minimum current intensity value of that participant. Next, the intensity of the electrical current was gradually increased for as long as it could be tolerated by the participant. The highest current that could be tolerated was identified as the maximum current intensity value, and all values were noted.

In the minimum intensity TENS application, electrical current was applied to participants at the minimum current intensity value. In the medium intensity TENS application, electrical current was applied to participants at the mean current intensity value (between the minimum and maximum current intensity). In the maximum intensity TENS application, electrical current was applied to participants at a value of 80% of the maximum current intensity value.

A dual channel (Model 120Z; Dual Channel, Tokyo, Japan) TENS device was used for the TENS application. For the individuals in both groups TENS was applied to the dorsal face of the right hand in continuous mode with asymmetric biphasic rectangular waveform, 100 Hz (hertz) pulse frequency, and 200 µs (microsecond) pulse width. The participants were instructed to minimize blinking and eye movements. The participants were seated in a dimly lit isolated room.

The stimuli and experimental procedures included four recording sessions: (1) Spontaneous EEG of the participants for 5 min (minute) with eyes open and 5 min with eyes closed. (2) Spontaneous EEG of the participants for 5 min with eyes open and 5 min with eyes closed during minimum intensity TENS application. (3) Spontaneous EEG of the participants for 5 min with eyes open and 5 min with eyes closed during medium intensity TENS application. (4) Spontaneous EEG of the participants for 5 min with eyes open and 5 min with eyes closed during maximum intensity TENS application.

### EEG recording

Electroencephalogram was recorded from 32 channels by using the BrainAmp amplifier and Brain Vision Recorder Software (Brainproducts, Munich, Germany). The EEG signals were amplified via BrainAmp with band limits of 0.01–250 Hz and sampled at 500 Hz. The electrodes (Ag/AgCl) were placed according to the International 10–20 system on the elastic cap. In addition, two physically linked earlobe electrodes (A1 + A2) were used as references. The electrooculogram was recorded from both the medial upper and lateral orbital rim of the left eye. Impedance values of electrodes were below 10 kΩ (kiloohm).

### EEG data processing

Brain Vision Analyzer 2.1 Software (Brainproducts, Munich, Germany) was used for EEG data processing. EEG recordings from F3, F4, C3, C4, CP3, CP4, P3, P4, P7, P8, O1 and O2 channels were analyzed. These electrodes were selected according to a careful inspection of grand averages. In our study, the spontaneous EEG analyses were performed separately for “eyes open” and “eyes closed” conditions. Raw data were divided into 1 s epochs, both “eyes open” and “eyes closed” conditions. Epochs containing artifacts such as eye movement, blinking, and muscle movement were removed off-line by a senior EEG researcher. In the next step, power spectrum analysis was performed using Fast Fourier Transform (FFT) (with 0.9 Hz maximum resolution; 10% Hanning window). The FFT power values of all epochs were averaged for each session. In the present study, the frequency of interest was alpha (8–13 Hz) because a major change in the alpha frequency band was detected between the young and elderly groups by the careful visual inspection of the grand averages of FFTs. Based on the results of previous studies, alpha frequency was determined as 8–13 Hz (Debener et al., 2000; Tran, Craig & McIsaac, 2001; Başar et al., 2001; Tran et al., 2004; Ghione et al., 2005; Lörincz, Crunelli & Hughes, 2008; Jensen & Mazaheri, 2010). The maximum alpha frequency peak value was measured in the 8–13 Hz alpha frequency range for each electrode of each participant (Başar et al., 2012; Benwell et al., 2019). This value was determined as the maximum individual alpha frequency value for the statistical analysis.

### Statistical analysis

SPSS version 22.0 was used for the statistical analysis. Three different repeated measures of analysis of variance (ANOVA) were applied for the statistical analysis of the spontaneous EEG. In order to compare group differences, young and elderly subject groups were identified as between-subject factor in the first analysis. Statistical analysis of HY and HEs was also separately analyzed as second and third ANOVA designs, in order to focus on the effects of the minimum, moderate and maximum electrical intensity levels separately in both groups. In the first ANOVA design, Between-Group Analysis was performed between “young” and “elderly” groups, and within-group analysis was performed for Eyes (two levels: eyes open and eyes closed), Condition (four levels: spontaneous EEG without TENS, spontaneous EEG with minimum intensity TENS, spontaneous EEG with medium intensity TENS, and spontaneous EEG with maximum intensity TENS), Location (six levels: frontal (F3–F4), central (C3–C4), centro-parietal (CP3–CP4), parietal-1 (P3–P4), parietal-2 (P7–P8) and occipital (O1–O2)), and Hemisphere (two levels: left and right). In the second ANOVA design, the statistical analysis of the HY was performed. In that analysis, repeated measures of ANOVA included Eyes (two levels: eyes open and eyes closed); Condition (four levels: no TENS, minimum intensity TENS, medium intensity TENS, and maximum intensity TENS); Location (six levels: frontal (F3–F4), central (C3–C4), centroparietal (CP3–CP4), parietal-1 (P3–P4), parietal-2 (P7–P8) and occipital (O1–O2)); Hemisphere (two levels: left and right) as within-subject factors. In the third ANOVA design, the statistical analysis of the HE was performed. In the analysis of the HE, repeated measures of ANOVA included Eyes (two levels: eyes open and eyes closed); Condition (four levels: no TENS, minimum intensity TENS, medium intensity TENS, and maximum intensity TENS); Location (six levels: frontal (F3–F4), central (C3–C4), centroparietal (CP3–CP4), parietal-1 (P3–P4), parietal-2 (P7–P8) and occipital (O1–O2)); Hemisphere (two levels: left and right) as within-subject factors. Greenhouse–Geisser corrected *p*-values were reported. Post hoc comparisons were performed by the Bonferroni test. Furthermore, the Non-parametric Mann–Whitney-*U* test was used to test the significance of the difference between the groups’ electrical current intensity values. The significance level was determined as *p* < 0.05 for all comparisons. To determine sample size, the power analysis was performed using G-Power 3.1.9.4 software (Faul et al., 2009). A priori power calculation was performed using repeated measures ANOVA within-between interaction (*d* = 0.23, α = 0.05, and power = 0.80).

## Results

### Electrophysiological results

#### Between-group analysis

There was no statistically significant difference between the two groups (*F*_(df = 1, 27)_ = 1.185; *p* = 0.286; ηp^2^ = 0.042).

**Figure 1:**
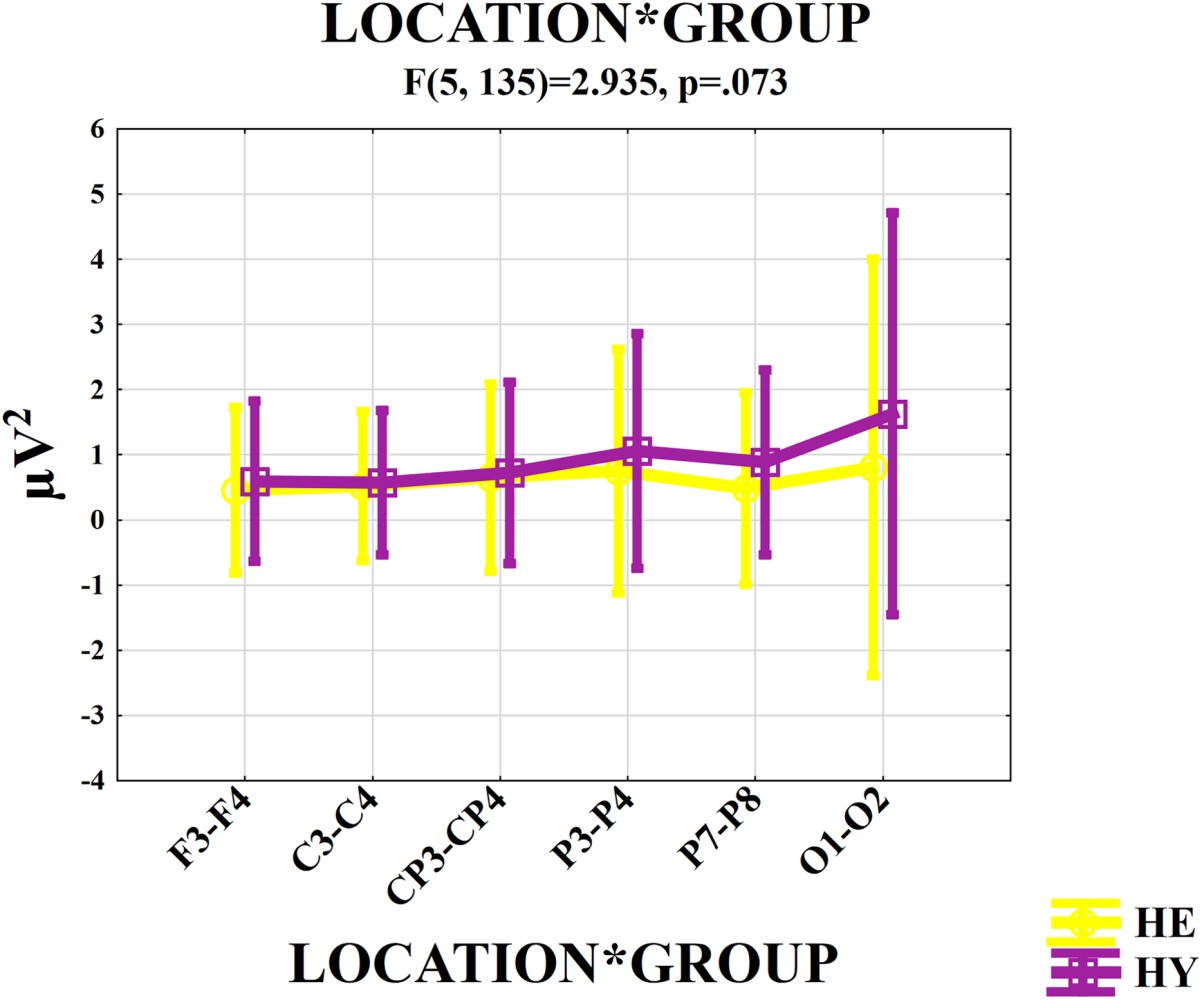
Graph of mean maximum spectral alpha power values in all locations for the healthy young group and healthy elderly group. Healthy elderly subjects (yellow line) are represented by HE, Healthy young subjects (purple line) are represented by HY. Frontal locations are represented by F3–F4, central locations are represented by C3–C4, centro-parietal locations are represented by CP3–CP4, parietal-1 locations are represented are P3–P4, parietal-2 locations are represented by P7–P8, and occipital locations are represented by O1–O2.

The locationXgroup interaction was close to the significance level (*F*_(df = 5, 135)_ = 2.935; *p* = 0.073; ηp^2^ = 0.098). As seen in Fig. 1, alpha activity in the occipital and parietal region of the HY was higher than in the HE.

The location effect was statistically significant (*F*_(df = 5, 135)_ = 9.627; *p* = 0.001; ηp^2^ = 0.263). Post-hoc comparisons showed that occipital locations elicited higher alpha activity than did the frontal, central, centro-parietal, and parietal-2 locations (*p* < 0.0001 for frontal, central, centro-parietal, and parietal-2). Parietal-1 locations elicited higher alpha activity than did the frontal and central locations (*p* < 0.05).

The eyes effect was statistically significant (*F*_(df = 1, 27)_ = 11.108; *p* = 0.003; ηp^2^ = 0.291). The eyesXhemisphereXgroup interaction was statistically significant (*F*_(df = 1, 27)_ = 4.476; *p* = 0.044; ηp^2^ = 0.142).

#### Analysis of the HY

**Figure 2:**
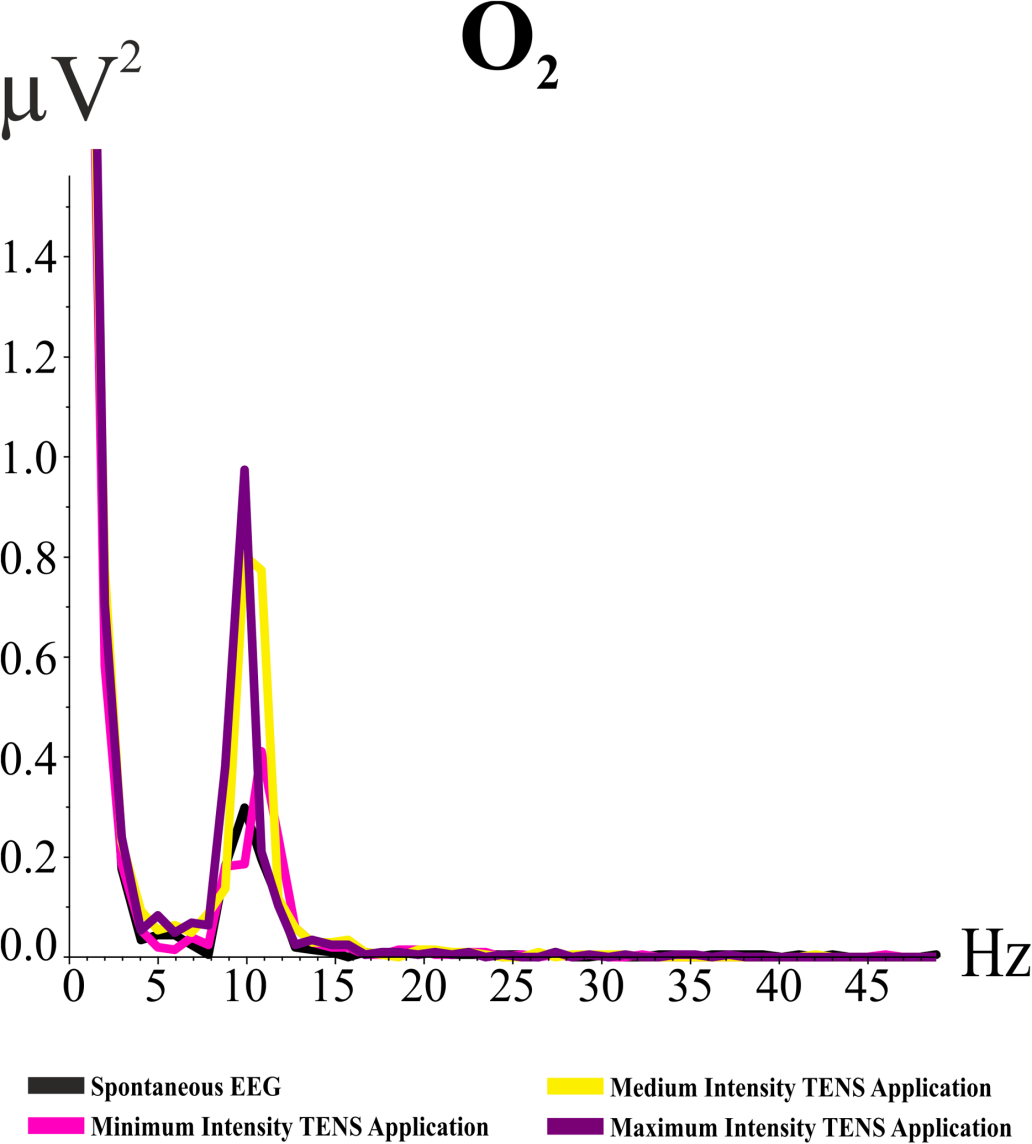
Grand averages of power spectrum analysis for O_2_ electrode band for the “eyes open” condition. The *Y*-axis indicates the values of the power spectrum, and *X*-axis indicates frequency values. The black line refers to spontaneous EEG alpha activity, the pink line refers to the alpha activity during the minimum intensity TENS application, the yellow line refers to the alpha activity during the medium intensity TENS application, and the purple line refers to the alpha activity during the maximum intensity TENS application.

The condition effect was statistically significant in the HY (*F*_(df = 3, 42)_ = 3.803; *p* = 0.035; ηp^2^ = 0.214). Post hoc comparisons showed that the alpha activity during the maximum intensity TENS application (*M:* 1.06, SE: 0.23) was higher than spontaneous EEG alpha activity (*M:* 0.76, SE: 0.17) for the HY (*p* < 0.0001). Figure 2 illustrates the grand averages of the power spectrum for the O2 electrode band in the HY during the “eyes open” condition. As illustrated in Fig. 2, the value of the alpha frequency is 0.3 µV^2^ when there was no TENS application. Alpha activity increased with the minimum intensity TENS application and was higher at medium intensity than minimum intensity. The highest alpha activity was evoked by the maximum intensity TENS application. Thus, alpha activity increased gradually in the HY.

The location effect was statistically significant in the HY (*F*_(df = 5, 70)_ = 7.748; *p* = 0.006; ηp^2^ = 0.356). Post-hoc comparisons showed that occipital locations elicited higher alpha activity than did the frontal, central, centro-parietal, and parietal-2 locations (*p* < 0.0001 for frontal, central, centro-parietal, and parietal-2).

The eyes effect was near to the significant level for the HY (*F*_(df = 1, 14)_ = 4.064; *p* = 0.063; ηp^2^ = 0.225).

#### Analysis of the HE

**Figure 3:**
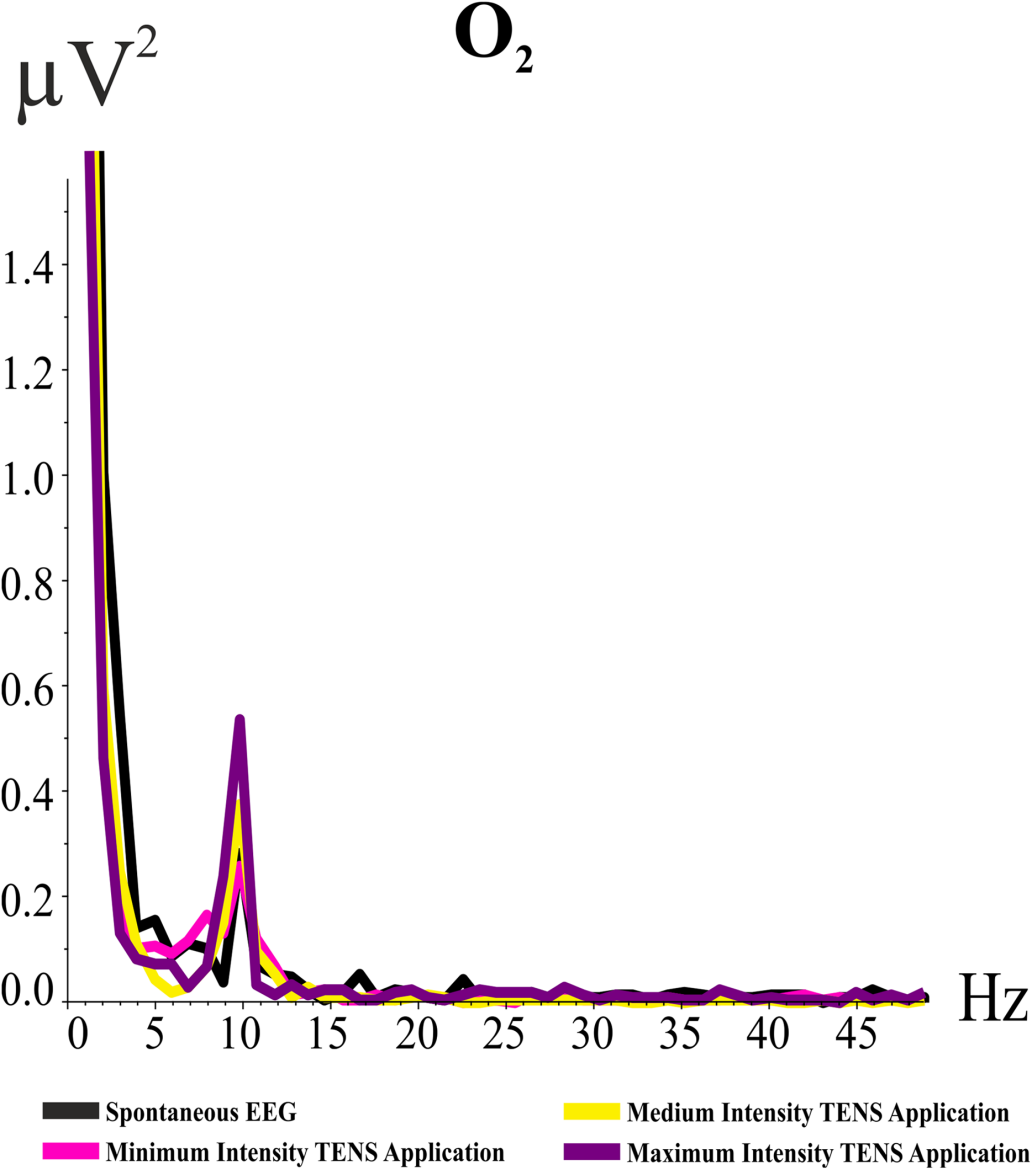
Grand averages of power spectrum analysis for O_2_ electrode band for the “eyes open” condition. The *Y*-axis indicates the values of the power spectrum, and *X*-axis indicates frequency values. The black line refers to spontaneous EEG alpha activity, the pink line refers to the alpha activity during the minimum intensity TENS application, the yellow line refers to the alpha activity during the medium intensity TENS application, and the purple line refers to the alpha activity during the maximum intensity TENS application.

The condition effect was not significant in the HE (*F*_(df = 3, 39)_ = 0.273; *p* = 0.705; ηp^2^ = 0.021). Figure 3 illustrates the grand averages of the power spectrum for the O_2_ electrode band for the HE in the “eyes open” condition. As illustrated in Fig. 3, the value of the alpha frequency is 0.3 µV^2^ when there was no TENS application. Alpha activity decreased in the minimum intensity TENS application and then alpha activity increased with the medium intensity TENS application. The increase in alpha activity with maximum intensity TENS application was higher than with medium intensity TENS application. Alpha activity did not gradually increase as it did in the HY.

The location effect was close to the significant level for the HE (*F*_(df = 5, 65)_ = 3.014; *p* = 0.088; ηp^2^ = 0.188). The eyes effect was statistically significant in the HE (*F*_(df = 1, 13)_ = 10.388; *p* = 0.007; ηp^2^ = 0.444). Furthermore, the eyesXlocationXhemisphere interaction was statistically significant for the HE (*F*_(df = 5, 65)_ = 3.150; *p* = 0.034; ηp^2^ = 0.195).

### TENS current intensity results

The current intensity effect was statistically significant between groups for three electrical current values (*p* < 0.05). TENS current intensity values perceived by healthy elderly subjects were higher than in healthy young subjects. Significant differences for TENS current intensity values are shown in Table 2.

**Table 2:** Significant differences for TENS current intensity values perceived by participants.

	*p*
Minimum intensity	0.041
Medium intensity	0.004
Maximum intensity	0.014

## Discussion

In the present study, we investigated the effects of TENS on EEG alpha activity. TENS was applied to healthy young and elderly subjects at different current intensities, gradually changing from low intensity to high current intensity. The present study aimed to find out the effect of TENS on EEG alpha oscillations in both young and elderly subjects. Since the alpha activity was increased during sensory stimulation in previous studies (Fu et al., 2001; Haegens et al., 2010; Grent-’t-Jong et al., 2011; Başar, 2012) we performed the analysis in the alpha frequency band. The previous literature showed decreased sensory functions in the elderly group (Kolev et al., 2002; Yordanova, Kolev & Başar, 1998; Dushanova & Christov, 2014). Therefore we expected reduced alpha activity in the elderly healthy subjects in comparison with young healthy subjects in response to TENS application. The results of the present study clearly showed the effect of TENS application on alpha activity and the effects of aging on the sensory perception of this application. The healthy young subjects had increased alpha activity during TENS application. Furthermore, there was a gradual increase in alpha activity with increasing current intensity in healthy young subjects. However, there was no increase in the alpha activity during TENS application in the healthy elderly subjects. In the healthy young subjects, alpha activity was higher in occipital and parietal locations than in other locations. The alpha activity of the occipital and parietal regions in the HY was higher than in the HE. The values of the current intensity perceived by healthy elderly subjects were higher than in healthy young subjects.

The present study presents that healthy young subjects had increased alpha activity during TENS application. Furthermore, there was a gradual increase in alpha activity with increasing current intensity in healthy young subjects. However, there was no increase in the alpha activity during TENS application in the healthy elderly subjects. These findings are in accordance with the literature. Previous studies investigated the pain-related EEG responses and brains’ electrical activity during in response to changing of somatosensory stimulus intensity (Chen & Rappelsberger, 1994; Chang et al., 2003; Babiloni et al., 2006; Dowman, Rissacher & Schuckers, 2008; Egsgaard, Wang & Arendt-Nielsen, 2009; Nir et al., 2012; Tiemann et al., 2015; Li et al., 2016; Misra et al., 2017; Wang et al., 2019; Vuckovic et al., 2019). The effect of TENS which was somatosensory stimulus changes according to the intensity of the electrical stimulus (low/high-intensity stimulation) (Johnson, Oxberry & Simpson, 2008; Robb et al., 2008). Most of these studies reported that especially alpha frequency was influenced to changing of the intensity of pain perception (Le Pera et al., 2000; Chang et al., 2001, 2003; Babiloni et al., 2006; Egsgaard, Wang & Arendt-Nielsen, 2009; Tiemann et al., 2015; Li et al., 2016; Tu et al., 2016; Wang et al., 2019; Vuckovic et al., 2019). Previous studies suggested that alpha activity was associated with various sensory functions and was influenced by sensorial stimulation (Le Pera et al., 2000; Schürmann & Başar, 2001; Başar, 2012). In the literature, EEG-alpha oscillations indicated as a marker of the responsiveness of the central nervous system to sensory stimulation (Başar & Güntekin, 2012). Thalamic nuclei are the main determinants of alpha activity in the cortical brain. Activation of the thalamus triggers alpha activity (Klimesch, Sauseng & Hanslmayr, 2007). In addition, previous studies indicated that the alpha frequency had increased due to inhibition (Jensen & Mazaheri, 2010; Klimesch, 2011; Roux & Uhlhaas, 2014). Le Pera et al. (2000) suggested that the increase of alpha activity could be due to synchronization in the somatosensory area. Furthermore, this increase could be due to the decrease in cellular excitability and the function of pain suppression. According to Klimesch, Sauseng & Hanslmayr (2007) an increase in the amplitude of the alpha frequency (event-related synchronization/ERS) reflects inhibition processes and plays a role in the inhibitory control system. Brain electrical oscillations are controlled by neurotransmitters. The releasing of these neurotransmitters affects brain electrical activity. Therefore, both the thalamus and neurotransmitters are important for sensory processes. Pain sensation starts from nociceptive receptors and travels from the medulla spinalis to the thalamus by spinothalamic tracts. Finally, pain sensation travels to the somatosensory cortical area of the brain by the thalamocortical fibers. Therefore, the thalamus, thalamocortical fibers, spinothalamic tracts, and nociceptors are important structures in the transmission and perception of pain (Wickremaratchi & Llewelyn, 2006; Price, 2002). TENS is used commonly in chronic and acute pain conditions. There are effective mechanisms of TENS at the central nervous system level, peripheral nervous system level and receptor level. At the central nervous system level, TENS effects inhibitory systems. In the peripheral nervous system level, TENS reduces the conduction of the sensory information to the central nervous system by afferent fibers, thereby decreasing the excitability of nociceptors. At the receptor level, TENS increases the release of neurotransmitters such as serotonin and GABA, thereby activating inhibitory mechanisms. In summary, TENS reduces the excitability of both the central nervous system and the peripheral nervous system by activating inhibitory mechanisms, thereby reducing the perception of the nociceptive stimuli (i.e., TENS) or pain (Vance et al., 2014). In the present study, TENS was used as somatosensory stimuli. When the action mechanisms of TENS are investigated, it can be observed that inhibitor neurons played a role consistent with both the gate control theory and the endogenous opioid system theory. Inhibition was provided in the spinothalamic pathway by inhibitory neurons (Le Pera et al., 2000; Klimesch, Sauseng & Hanslmayr, 2007; Eti, 2007). Therefore, the spinothalamic pathway and thalamus are important in the inhibitory mechanism. The maximum alpha frequency peak which was one of the alpha activity indices reflects inhibition of neuronal activation (Klimesch, Sauseng & Hanslmayr, 2007; Bazanova & Vernon, 2014; Bazanova, Auer & Sapina, 2018). It can be inferred that the increase in alpha activity of the spontaneous EEG in response to TENS application is due to inhibition of neuronal activation and these inhibitory neurons taking part in the action mechanism of TENS. This may indicate that TENS is used as somatosensory stimuli and that both TENS and alpha frequency peak are related to the inhibitory processing. It can be assumed that TENS induces inhibition which increases alpha activity. Many studies reported age-related changes in thickness and volume of the gray matter and hyperintensities of the white matter (Haga et al., 2009; Raininko & Mattsson, 2010; Maudsley, Govind & Arheart, 2012; Hedden et al., 2016; Ding et al., 2016). The brain metabolites are associated with the neuronal metabolic activity, energy metabolism, neuronal integrity, and/or function of the neurotransmitters. It has been reported that the brain metabolite concentrations change along with aging (Grachev & Apkarian, 2001; Barker et al., 2010; Ding et al., 2016). These studies demonstrated a decrease in gray matter volume and the brain water content decreased along with aging. In addition, it was reported that increased total cranial cerebrospinal fluid volume along with aging. The brain metabolites (N-acetyl-aspartate and the fractional volume) decreased in the gray matter and some brain metabolites in cerebral white matter (choline, total creatine, and myoinositol) were increased along with aging (Grant et al., 1987; Chang et al., 1996; Leary et al., 2000; Brooks et al., 2001; Allen et al., 2005; Raininko & Mattsson, 2010; Maudsley, Govind & Arheart, 2012; Reyngoudt et al., 2012; Hedden et al., 2016). Decreased brain metabolites of the gray matter could be related to a decrease in neuronal volume, neuronal density or metabolic activity. Increased brain metabolites of the white matter are related to altered energy metabolism. Previous studies suggested that, along with aging, there was a reduction in the number of nerve cells in the brain and the number of receptors under the skin (Nalbant, 2008; Kalisch, Tegenthoff & Dinse, 2008; Skedung et al., 2018). Most of the studies showed that functional and structural changes in the nerve fibers, thalamus, and cortex occur with aging (Sato, Sato & Suzuki, 1985; Katzman, 1992; Verdú et al., 2000; Kemp et al., 2014). The decrease in alpha activity in elderly subjects compared to young subjects could be a result of the decrease in the number of nerve cells in the brain, a reduction in neuronal volume, number or metabolic activity, altered energy metabolism, and a change of the inhibition mechanism of the cortex, thalamus, and spinothalamic pathway that occurs with aging.

In the present study, the alpha activity of the occipital and parietal locations was higher in comparison with other locations in healthy young subjects. The present study confirmed the results of previous studies. These earlier studies demonstrated that spontaneous EEG alpha activity is the major rhythm of the posterior cortical areas in healthy individuals and that alpha activity is especially dominant in the occipital region (Başar et al., 1997; Yordanova, Kolev & Başar, 1998). Furthermore, both lesion and fMRI studies have shown that some cortical regions play a role in both the processing and perception of the somatosensory stimuli. These cortical areas were the primary somatosensory, secondary somatosensory, and posterior parietal cortical areas (Price, 2002; Porro et al., 2002).

In the results of the present study, the alpha activity of the occipital and parietal region in the HY was higher than the HE. The decrease of the alpha activity in posterior cortical areas reflects the impairment in the inhibition processing (suppressed of the non-related information) (Min & Herrmann, 2007; Bazanova, Auer & Sapina, 2018). Lesion studies have demonstrated that some cortical regions play a role in the processing of painful stimuli. In the case of damage to the primary somatosensory and secondary somatosensory cortical areas, perception of the pain sensation is impaired (Price, 2002). Most of the fMRI studies indicated that painful stimuli application results in activation in the thalamus, primary somatosensory, secondary somatosensory, and posterior parietal cortical areas (Porro et al., 2002). Furthermore, PET scan studies showed that there was activation in the sensorial cortex, and other parts of parietal and occipital cortical area during the application of painful stimuli (Aydın, 2002). In the literature, there are many studies showing that EEG oscillatory activity in the human brain changes from birth to senility (Niedermeyer, 1997; Polich, 1997; Yordanova, Kolev & Başar, 1998; Kolev et al., 2002; Rossini et al., 2007; Başar, 2012). Previous studies indicated that alpha power decreased, especially in the occipital and parietal locations in elderly subjects in comparison with young healthy subjects (Klass & Brenner, 1995; Polich, 1997; Rossini et al., 2007; Dushanova & Christov, 2014). In accordance with the literature, the present study showed that the alpha activity of healthy elderly subjects had decreased in the occipital and parietal locations. This result could be due to both TENS, which when used as somatosensory stimuli increased the alpha activity of the parietal and occipital locations corresponding to sensory areas, and alpha activity that decreased depending on changes in the nervous system along with aging.

In the present study, the values of the current intensity perceived by healthy elderly subjects were higher than in healthy young subjects. The central nervous system is important in the perception of pain. Previous studies showed that the central nervous system activation associated with the processing of painful, noxious, and/or somatosensory stimuli had decreased in elderly subjects (Wickremaratchi & Llewelyn, 2006). Along with aging, there are some changes in the central nervous system and the peripheral nervous system. The changes in the central nervous system include reduction of the weight of the human brain, a loss of cortical neurons, a reduction in the concentration of receptors (dopamine, acetylcholine, noradrenaline, GABA etc.), and a decrease in the myelin and intracellular enzymes. These changes in the central nervous system may cause slowing in the processing of sensory stimuli (Victor & Ropper, 2001; Katzman & Terry, 1983). The changes in the peripheral nervous system include a decrease in the density of myelinated fibers (Corbin & Gardner, 1937), a degeneration in the distal parts of the afferent fibers (Ohnishi et al., 1976), a reduction in the total fiber number of nerves and in the density of nerves (Swallow, 1966; O’Sullivan & Swallow, 1968; Tohgi, Tsukagoshi & Toyokura, 1977; Chang, Lin & Hsieh, 2004). The nerve conduction studies demonstrated that, along with aging, there was a decrease in the speed of sensory conduction, and in the amplitude of the action potential initiated by sensory stimulation (Verdú et al., 2000). A retrospective study showed a correlation between aging and an amplitude of the action potential resulting from sensory stimulation. Furthermore, a negative relationship between aging and the speed of sensory conduction was also reported by Rivner, Swift & Malik (2001). Most of the studies demonstrated decreased perception of pain due to aging, and that elderly subjects had higher sensory and pain thresholds than did young subjects (Neri & Agazzani, 1984; Wickremaratchi & Llewelyn, 2006; De Jesus Guirro, De Oliveira Guirro & De Sousa, 2015; Lautenbacher et al., 2017). The present study confirmed the previous studies; elderly subjects had a higher perception threshold value for electrical stimuli applied by TENS in comparison with young subjects. The higher threshold values of elderly subjects could be due to a loss of the afferent axons and changes in their subcutaneous receptors.

The menstrual cycle in the female subjects was reported to be essential for the sensation of pain. The influence of the menstrual cycle and using oral contraceptives on the EEG alpha activity was investigated in relation to hormonal changes. Some studies demonstrated that menstrual cycle and use of the oral contraceptives could effect the nervous thresholds of women (Barbosa, Guirro & Nunes, 2013; Brötzner et al., 2014; Bazanova et al., 2014; Bazanova, Nikolenko & Barry, 2017). One of the limitations of the present study is that the menstrual cycle of the young female subjects was not evaluated. In future studies, the effect of the menstrual cycle and the use of oral contraceptives on the spontaneous EEG activity during TENS application should also be studied in women. Another limitation of the present study is the application of the TENS. We have preferred to apply the different intensities of TENS in a serial design rather than a random design. We have preferred to apply the application in a serial design in order to be standard for all groups of subjects. However, the same procedure should also be studied in a random design in order to be sure that the healthy young elderly subjects had increased alpha power with an increased TENS intensity. One of the other limitations of the present study is the individual alpha frequency was not analyzed. The individual alpha peak frequency is one of the important alpha indices that is showed alpha activity and varies inter-individually and intra-individually (Bazanova & Vernon, 2014). We have performed the analysis of the amplitude of the power spectrum in the classical 8–13 Hz band which is very commonly used in the literature. However, in order to enlighten the research for future studies, individual alpha frequency should also be studied.

## Conclusions

In conclusion, TENS application in the different current intensities resulted in increased alpha activity in the somatosensory and occipital cortical areas in young healthy subjects. The increased alpha activity reflects inhibition of neuronal activation in healthy young individuals. The decreased alpha activity in posterior areas in comparison to young individuals reflects the impairment in the inhibition processing in elderly individuals. To our knowledge, the present study is one of the first studies that examine the effect of TENS on spontaneous EEG in healthy young and healthy elderly subjects. These results are relevant in the literature in terms of establishing a standardization regarding healthy young and healthy elderly individuals. The results of the present study, obtained through the investigation of healthy subjects, may be used as a standard in an examination of different pathologies involving sensory impairment.

## Additional Information and Declarations

### Data Availability

The following information was supplied regarding data availability: The raw data is available at Figshare: Yıldırım, Ebru; Güntekin, Bahar; Hanoğu, Lütfü; Algun, Candan (2019): EEG alpha activity in response to Transcutaneous Electrical Nervous Stimulation in healthy young subjects and in the healthy elderly subjects. figshare. Dataset. DOI 10.6084/m9.figshare.11347247.v1.
